# The Sulforaphane and pyridoxamine supplementation normalize endothelial dysfunction associated with type 2 diabetes

**DOI:** 10.1038/s41598-017-14733-x

**Published:** 2017-10-30

**Authors:** Ana Pereira, Rosa Fernandes, Joana Crisóstomo, Raquel M. Seiça, Cristina M. Sena

**Affiliations:** 1 Physiology, IBILI, Faculty of Medicine, University of Coimbra, Coimbra, Portugal; 2 Ophthalmology, IBILI, Faculty of Medicine, University of Coimbra, Coimbra, Portugal

## Abstract

In this study we investigate pyridoxamine (PM) and/or sulforaphane (SFN) as therapeutic interventions to determine whether activators of NFE2-related factor 2 (Nrf2) can be used in addition with inhibitors of advanced glycation end products (AGE) formation to attenuate oxidative stress and improve endothelial dysfunction in type 2 diabetes. Goto-kakizaki (GK) rats, an animal model of non-obese type 2 diabetes, were treated with or without PM and/or SFN during 8 weeks and compared with age-matched Wistar rats. At the end of the treatment, nitric oxide (NO)-dependent and independent vasorelaxation in isolated aorta and mesenteric arteries were evaluated. Metabolic profile, NO bioavailability and vascular oxidative stress, AGE and Nrf2 levels were also assessed. Diabetic GK rats presented significantly lower levels of Nrf2 and concomitantly exhibited higher levels of oxidative stress and endothelial dysfunction. PM and SFN as monotherapy were capable of significantly improving endothelial dysfunction in aorta and mesenteric arteries decreasing vascular oxidative damage, AGE and HbA1c levels. Furthermore, SFN + PM proved more effective reducing systemic free fatty acids levels, normalizing endothelial function, NO bioavailability and glycation in GK rats. Activators of Nrf2 can be used therapeutically in association with inhibitors of AGE and cross-linking formation to normalize endothelial dysfunction in type 2 diabetes.

## Introduction

Type 2 diabetes is associated with elevated levels of oxidative stress and glycation altering vascular function and is therefore considered a major risk factor underlying development of cardiovascular disease^1,2^. Many of the complications in diabetes are related to hyperglycaemia and increased generation of reactive oxygen species (ROS), which lead to endothelium dysfunction. Impaired endothelium-dependent relaxation to vasodilators such as acetylcholine is a common feature in both conduit and resistance arteries from experimental models of type 1 and type 2 diabetes^1,3–5^. We have previously shown that diabetic GK rats have increased oxidative stress and glycation, which leads to endothelial dysfunction^5,6^.

Progress in the treatment of diabetes has not been effective in decreasing vascular complications associated with the disease. Given the crucial role of ROS in endothelial function, considerable efforts have been made to discover therapies to reduce ROS in the vasculature.

Nuclear factor E2(erythroid-derived 2)-Related Factor-2 (Nrf2) is a transcription factor that plays a crucial role in the cellular protection against oxidative stress. Nrf2 is referred to as the “master regulator” of the antioxidant response^7,8^. Induction of endogenous antioxidant enzymes by activators of the Nrf2/antioxidant response element pathway may be an interesting approach to obtain sufficient levels of antioxidants and reduce oxidative stress. Dietary Nrf2 activators, such as the isothiocyanate sulforaphane (SFN) found in cruciferous vegetables, can increase antioxidant defenses, reduce blood pressure, inhibit pro-inflammatory signalling pathways in the kidney^9,10^ and prevent metabolic dysfunction in endothelial cells induced by hyperglycaemia^11^. In diabetic models, activation of Nrf2 lowers plasma glucose levels and reduces diabetes-related nephropathy in wild type but not in Nrf2 deficient mice^12^. Thus, the therapeutic potential of Nrf2 inducers in vascular disease is enormous in particular associated with long-term type 2 diabetes^8,13–15^.

Additionally, advanced glycation is accelerated under diabetic conditions leading to vascular complications. Several authors have proved that inhibition of advanced glycation end products (AGE) formation may be a therapeutic strategy to improve vascular complications in diabetes. Pyridoxamine (PM), a naturally occurring derivative of vitamin B6, has proved to be an effective inhibitor for protein glycation and lipoxidation^16^. PM has been found to inhibit the formation of AGE both *in vitro* and *in vivo* with potential benefits for the treatment of diabetic nephropathy and retinopathy^17^.

Sulforaphane and pyridoxamine have distinct mechanisms of action. Therefore, we hypothesized that either SFN, PM alone or in association could have beneficial effects on endothelium-dependent vascular reactivity, oxidative stress and metabolic parameters in Goto–Kakizaki (GK) rats, a model of type 2 diabetes. In parallel with the development of diabetes, lipid profile, oxidative stress, glycation and nitric oxide (NO) bioavailability were evaluated. Additionally, endothelial dependent and independent vascular sensitivity to acetylcholine (ACh) and sodium nitroprusside (SNP) were assessed in aorta and mesenteric arteries. Eight months old GK rats showed a long-term diabetic phenotype with endothelial dysfunction both at conduit and resistant arteries accompanied with increased oxidative stress when compared to Wistar rats. SFN and PM alone were capable of ameliorating NO-dependent vasorelaxation in isolated arteries. In association, SFN and PM normalized endothelial function both in aorta and mesenteric arteries through a mechanism that involves an increment in NO bioavailability and a decrement in oxidative stress and AGE levels. Altogether, these studies indicate that targeting different mechanisms underlying endothelial dysfunction such as inhibiting AGE formation (with PM) and promoting an increment in antioxidant defense systems (with SFN) could be considered as a useful tool for diabetic macrovascular complications due the simultaneous action on different mechanisms that ultimately lead to vascular damage.

## Results

### Animal characteristics

**Figure 1:**
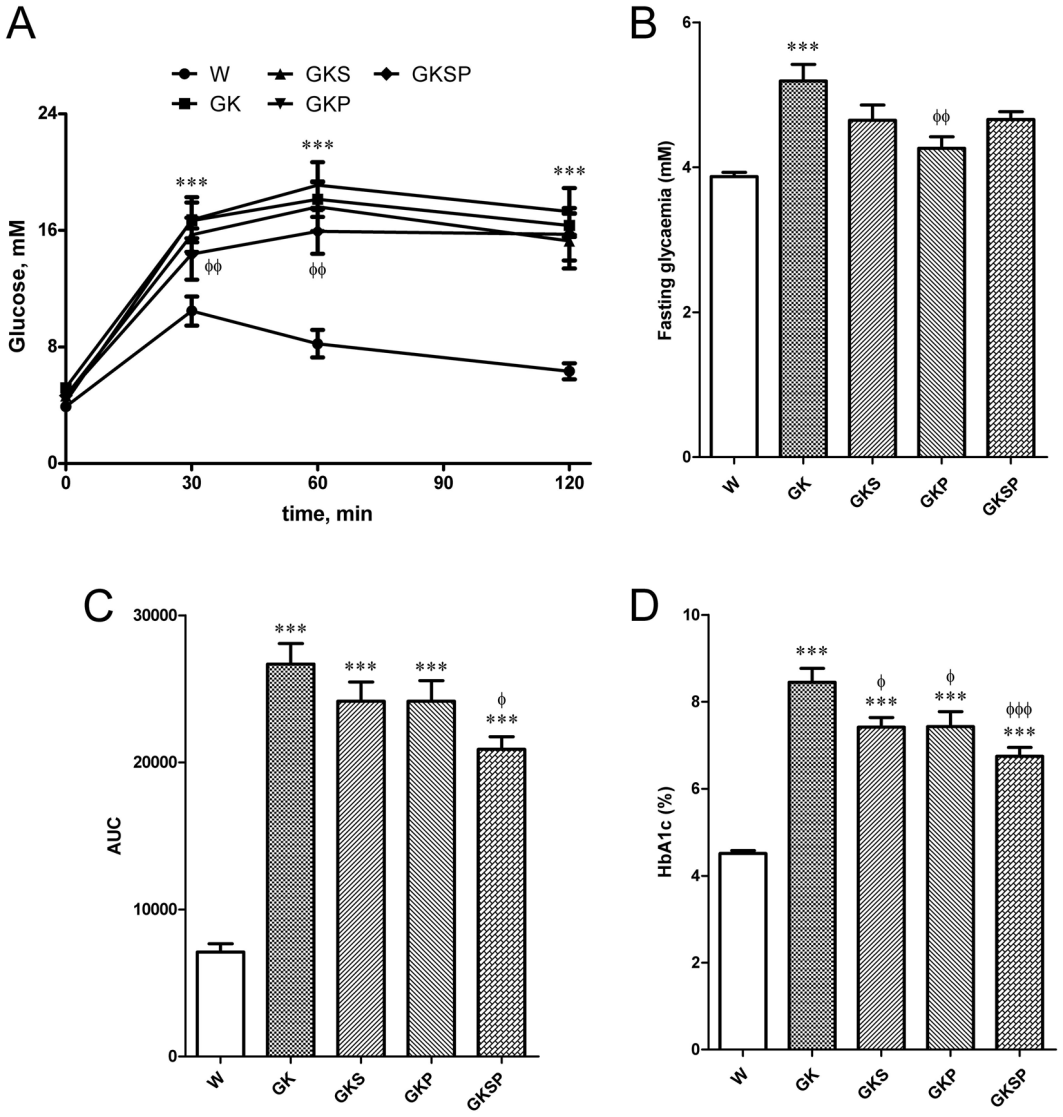
Effects of sulforaphane and pyridoxamine treatment on intraperitoneal glucose tolerance test (IPGTT; **A**), fasting glycaemia (**B**), the glucose area under the curve (AUC; **C**) and HbA1c levels (**D**) in 8 months old diabetic Goto-Kakizaki (GK) rats compared with nondiabetic Wistar (W) rats. **C**) The AUC of IPGTT curves was calculated to measure the degree of the glucose tolerance impairment. Data are expressed as mean ± SE (n = 12). ***P < 0.001 *vs* Wistar group; ^ϕ^P < 0.05, ^ϕϕ^P < 0.01, ^ϕϕϕ^P < 0.001 *vs* GK group.

The diabetic rats used in our experiments exhibited similar fasting plasma glucose levels at the beginning of the study. GK glycaemia evaluated before treatment (GK6m) was significantly higher when compared to age-matched non-diabetic Wistar rats (Fig. 1 supplement). Body weight was significantly lower in GK rats compared to age-matched Wistar rats and the different therapies did not significantly change this parameter (Table 1). In an intraperitoneal glucose tolerance test (IPGTT), GK exhibited marked glucose intolerance as compared with Wistar rats (Fig. 1A). Fasting glycaemia, the glucose area under the curve (AUC), HbA1c, total cholesterol and FFA were elevated in GK rats when compared to Wistar rats (Fig. 1B,C,D, Table 1). As previously^6^, GK rats exhibited normal non-HDL-cholesterol and triglycerides (Table 1). Treatment with SFN or PM alone did not significantly change glucose intolerance and the lipid profile evaluated (Fig. 1A,C, Table 1). Moreover PM significantly decreased fasting glucose and HbA1c levels (Fig. 1B,D). Indeed, all the therapeutic approaches were able to effectively reduce HbA1c levels in GK rats (Fig. 1D). Additionally, treatment with both SFN and PM for 8 weeks effectively reduced circulating concentrations of FFA in diabetic rats (Table 1) and also significantly ameliorated glucose intolerance (Fig. 1A,C).

**Table 1:** Body weight and lipid levels in 8 months old non-diabetic Wistar (W) and diabetic Goto-Kakizaki (GK) rats and in GK rats treated with sulforaphane (GKS), pyridoxamine (GKP) or sulforaphane and pyridoxamine (GKSP).

	W	GK	GKS	GKP	GKSP
Body weight (g)	478.5 ± 15.2	390 ± 5.7^***^	374.6 ± 5.3^***^	401.8 ± 6.6^***^	375 ± 9.3^***^
Total Cholesterol (mM)	2.07 ± 0.07	2.75 ± 0.07^**^	2.74 ± 0.14	2.53 ± 0.16^***^	2.71 ± 0.14 ^**^
Non-HDL cholesterol (mM)	0.87 ± 0.04	1.1 ± 0.06	1.02 ± 0.09	0.086 ± 0.08	1.14 ± 0.08
Triglycerides (mM)	0.94 ± 0.07	1.18 ± 0.1	1.04 ± 1.0	1.29 ± 0.07	1.11 ± 0.06
FFA (mM)	0.58 ± 0.02	1.21 ± 0.08^***^	0.97 ± 0.06 ^**^	0.99 ± 0.13^*^	0.73 ± 0.06^ϕϕϕ,§,#^

Data are expressed as mean±SE (n = 12 animals in each group).

^*^P < 0.05, ^**^P < 0.01, ^***^P < 0.001 *vs* W rats.

^ϕϕϕ^P < 0.001 *vs* GK rats.

^§^P < 0.05 *vs* GKS rats.

^#^P < 0.05 *vs* GKP.

### NO dependent vascular relaxation

**Figure 2:**
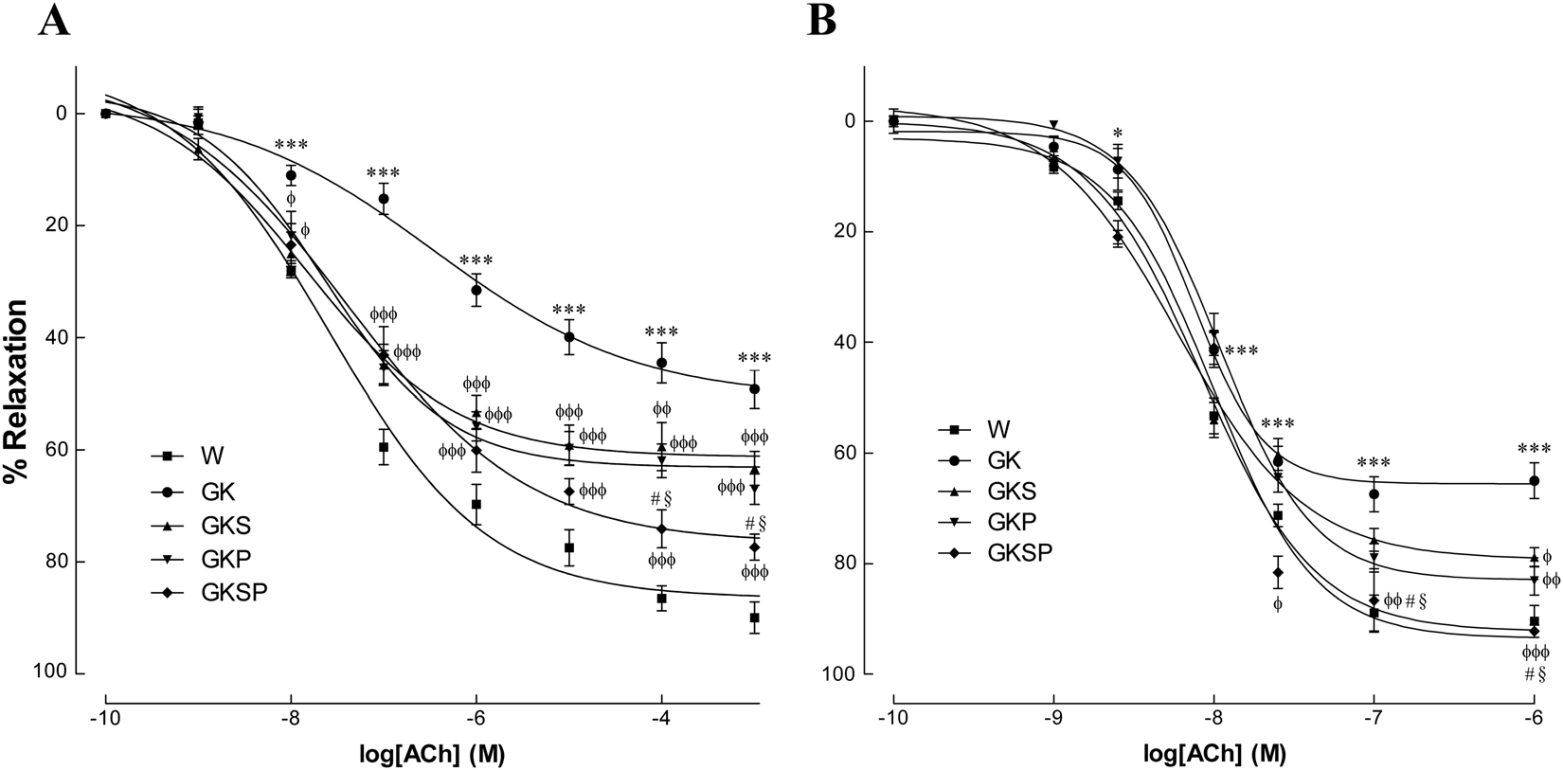
Effects of sulforaphane and pyridoxamine treatment on vasodilatory responses to acetylcholine in aorta (**A**) and mesenteric arteries (**B**) of GK rats compared with nondiabetic Wistar (W) rats. Data are expressed as mean ± SE (n = 12). *P < 0.05, ***P < 0.001 *vs* Wistar group; ^ϕ^P < 0.05, ^ϕϕ^P < 0.01, ^ϕϕϕ^P < 0.001 *vs* GK group; ^§^P < 0.05 *vs* GKS group; ^#^P < 0.05 *vs* GKP group.

**Figure 3:**
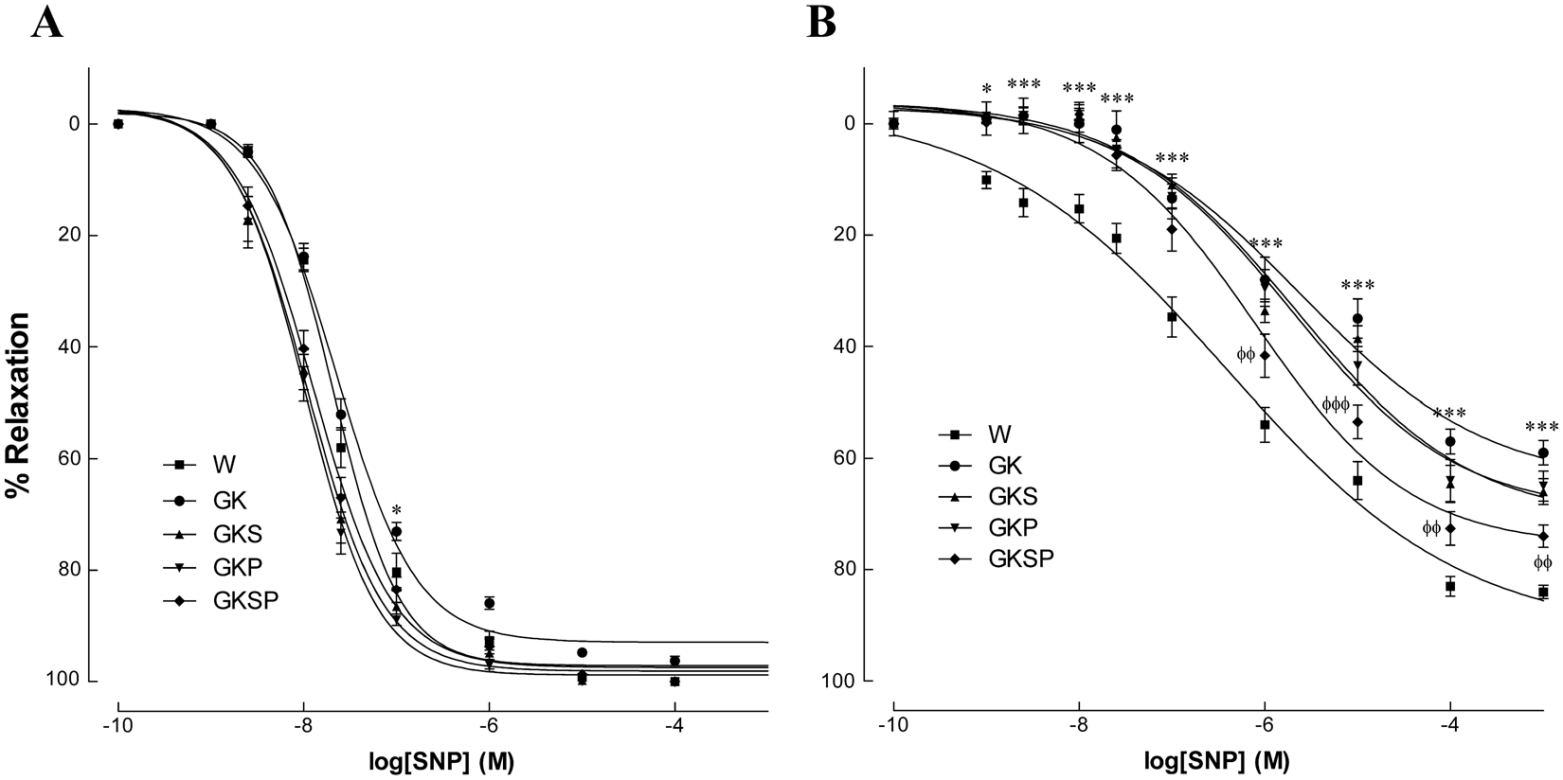
Effects of sulforaphane and pyridoxamine treatment on vasodilatory responses to sodium nitroprusside in aorta (**A**) and mesenteric arteries (**B**) of GK rats compared with nondiabetic Wistar (W) rats. Data are expressed as mean ± SE (n = 12). *P < 0.05, ****P < 0.001 *vs* Wistar group; ^ϕϕ^P < 0.01, ^ϕϕϕ^P < 0.001 *vs* GK group.

In 8 months old GK rats endothelium-mediated vascular relaxation of phenylephrine-precontracted aorta arterial rings in response to ACh was impaired compared with age-matched Wistar rats (Fig. 2A), but the endothelium-independent relaxations to sodium nitroprusside (SNP) were similar in both strains (Fig. 3A). Indeed, maximal endothelium-mediated relaxation of phenylephrine-precontracted aorta rings in response to ACh declined by 49% in diabetic rats (Fig. 2A). Preincubation of the arterial rings with the NOS inhibitor N-nitro-L-arginine- methyl ester (L-NAME) and the cyclooxygenase inhibitor indomethacin almost completely abolished relaxation by ACh in GK rats and Wistar rats (Fig. 2 supplement). The residual component due to other vasodilators is around 15% in our experimental conditions. In aorta, no differences on maximal relaxation were observed in the concentration-effect curves for SNP between the different groups (Fig. 3A). Maximal relaxation significantly improved in all GK treated groups with an improvement in vascular reactivity in response to ACh (Fig. 2A, Table 2). Treatment with SFN, PM or SFN + PM significantly improved endothelium-dependent vascular relaxation in aorta of diabetic rats (Fig. 2A, Table 2) This vasodilation is not sensitive to indomethacin and is blocked by L-NAME indicating that the mechanism is dependent on endogenous NO release (Fig. 2 supplement). Furthermore, an increment in SNP sensitivity was observed in all GK treated groups (Fig. 3A, Table 2). In mesenteric arteries, maximal endothelium-mediated relaxation of phenylephrine-precontracted rings in response to ACh declined by 36% in diabetic rats (Fig. 2B). Additionally, maximal relaxation in response to SNP declined by 39% in diabetic rats (Fig. 3B). Treatment with SFN, PM or SFN + PM was able to improve endothelial-dependent vasodilation (Fig. 2B, Table 3). Similarly, this effect is not sensitive to indomethacin and is inhibited by L-NAME (Fig. 2 supplement). Detailed data on maximal relaxations and EC_50_ values are summarized in Tables 2 and 3. These results indicated that treatment with SFN or PM alone improved endothelium-dependent vascular relaxation in both aorta and mesenteric arteries of diabetic GK rats. In association these therapies were able to normalize endothelium-mediated relaxation and improve SNP sensitivity in aorta and mesenteric arteries (Figs 2, 3).

**Table 2:** Maximal relaxation responses (%) and −logEC_50_ in isolated aorta arteries of 8 month-old variously treated spontaneously diabetic Goto-Kakizaki (GK) rats and age-matched non-diabetic Wistar rats.

	W	GK	GKS	GKP	GKSP
ACh					
pEC_50_	7.59 ± 0.1	6.47 ± 0.18^***^	7.8 ± 0.24^ϕϕϕ^	7.64 ± 0.13^ϕϕϕ^	7.4 ± 0.18^ϕϕ^
Maximal relaxation (%)	86.5 ± 3.8	50.7 ± 3.7^***^	63 ± 3.6^***,ϕϕϕ^	63.1 ± 2.7^***,ϕϕϕ^	74.3 ± 3.1 ^**,ϕϕϕ,§,#^
SNP					
pEC_50_	7.7 ± 0.03	7.6 ± 0.03	7.96 ± 0.05 ^***,ϕϕϕ^	7.98 ± 0.04^*,ϕϕϕ^	7.9 ± 0.04^*,ϕϕϕ^
Maximal relaxation (%)	97.1 ± 1.2	92.9 ± 1.4	98.1 ± 1.6	98.8 ± 1.4	97.5 ± 2.2

Data are expressed as mean±SE (n = 12 animals in each group). pEC_50_ values are presented as the negative logarithm (−logEC_50_) of concentration of the agonist.

^*^P < 0.05, ^**^P < 0.01, ^***^P < 0.001 *vs* W rats.

^ϕϕ^P < 0.01, ^ϕϕϕ^P < 0.001 *vs* GK rats.

^§^P < 0.05 *vs* GKS.

^#^P^ <^ 0.05 *vs* GKP.

**Table 3:** Maximal relaxation responses (%) and −logEC_50_ in isolated mesenteric arteries of 8 month-old variously treated spontaneously diabetic Goto-Kakizaki (GK) rats and matched non-diabetic Wistar rats.

	W	GK	GKS	GKP	GKSP
ACh					
pEC_50_	8.1 ± 0.04	8.1 ± 0.05	8.2 ± 0.04	7.9 ± 0.04	8.0 ± 0.04
Maximal relaxation (%)	92.3 ± 2.5	65.6 ± 2.5^***^	79.2 ± 2.4 ^***,ϕ^	83 ± 2.0^***,ϕϕ^	93.5 ± 2.4 ^***,ϕϕϕ,§,#^
SNP					
pEC_50_	6.4 ± 0.1	5.6 ± 0.2^***^	5.66 ± 0.14^***^	5.7 ± 0.14^***^	6.1 ± 0.07^***^
Maximal relaxation (%)	84 ± 1.2	60.8 ± 2.2	66 ± 2.3	65.3 ± 2.7	74 ± 2

Data are expressed as mean±SE (n = 12 animals in each group). pEC_50_ values are presented as the negative logarithm (−logEC_50_) of concentration of the agonist.

^***^P < 0.001 *vs* W rats.

^ϕ^P < 0.05, ^ϕϕ^P < 0.01, ^ϕϕϕ^P < 0.01 *vs* GK rats.

^§^P < 0.05 vs GKS.

^#^P < 0.05 vs GKP.

### Oxidative stress in the vascular wall

**Figure 4:**
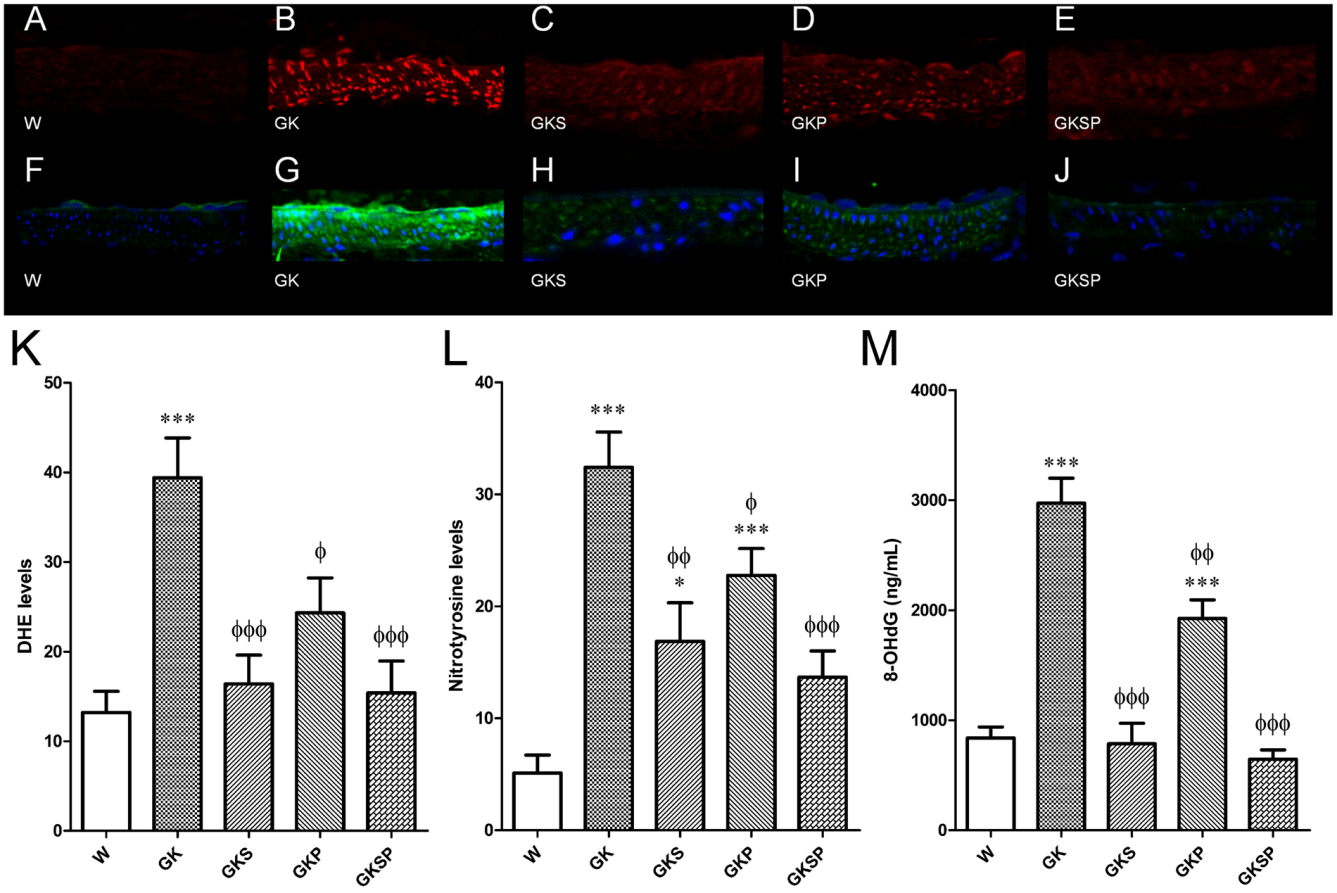
Effects of sulforaphane and pyridoxamine treatment on systemic and vascular oxidative stress in GK rats compared with nondiabetic Wistar (W) rats. Representative DHE-stained aorta artery sections reflect O_2_·^–^ production with the different treatments (**A**–**E**). The endothelium is facing up in all layers. At identical settings, fluorescence in diabetic GK (B) was markedly increased compared with normal vessel (Wistar, **A**). Note the increased fluorescence reflecting O_2_·^−^ levels in the endothelium, intima, and media of GK aorta. DHE fluorescence decreased in the diabetic GK rats treated with SFN (GKS, **C**), PM (GKP, **D**) and SFN + PM (GKSP, **E**). Panel (K) contains quantification of the fluorescence ethidium signal in the different groups of arteries. Representative mesenteric sections showing nitrotyrosine staining in nondiabetic Wistar (**F**), diabetic GK (**G**), and diabetic GK rats treated with SFN (GKS, **H**), PM (GKP, **I**) and SFN + PM (GKSP, **J**). Panel (**L**) contains quantification of the green fluorescence in the different groups of arteries. (**M**) Urinary 8-hydroxydeoxyguanosine (8-OHdG) levels in the different groups of rats. Data are expressed as mean ± SE (n = 12). *P < 0.05, ***P < 0.001 *vs* Wistar group; ^ϕ^P < 0.05, ^ϕϕ^P < 0.01, ^ϕϕϕ^P < 0.001 *vs* GK group.

We determined the potential impact of SFN, PM or both on the oxidative stress in the diabetic vasculature. Interestingly, diabetes induced a 3-fold increase in superoxide production in diabetic aorta (p < 0.001; Fig. 4B,K). The density of dihydroethidium (DHE) was significantly decreased in the aorta of diabetic rats treated with SFN, PM and SFN + PM compared to GK rats (Fig. 4C,D,E,K). Additionally, diabetic GK rats also had increased immunoreactive nitrotyrosine levels in their mesenteric arteries (p < 0.001; Fig. 4G,L) and treatment with SFN, PM or both significantly decreased these levels (Fig. 4H,I,J,L). Thus, all therapies significantly reduced vascular oxidative damage in diabetic GK rats both in aorta and mesenteric arteries. Accordingly, urinary levels of 8-hydroxy-2′-deoxyguanosine (8-OHdG) were significantly higher in GK rats, when compared to age-matched Wistar rats (Fig. 4M). Treatment with SFN, PM or both for 8 weeks to GK rats significantly reduced 8-OHdG levels (Fig. 4M).

### pVASP and total VASP in aorta

**Figure 5:**
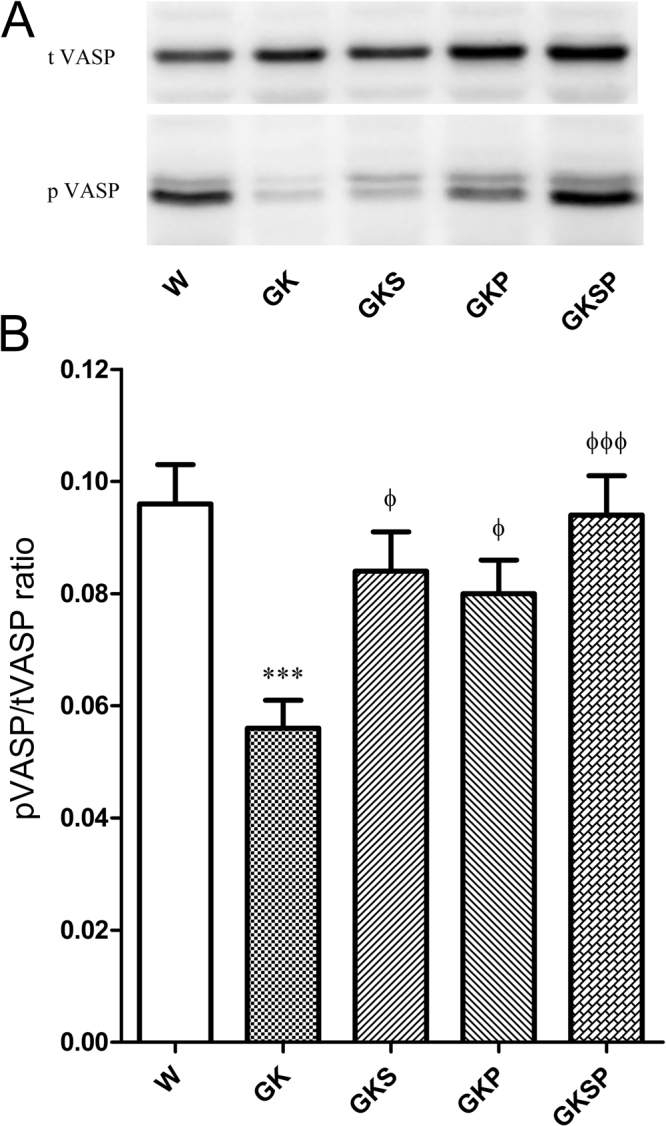
Effects of sulforaphane and pyridoxamine treatment on pVASP and tVASP expression levels. To examine NO-cGMP signal activation, total vasodilator-stimulated phosphoprotein (t VASP) and phosphorylated (Ser239) VASP (pVASP) expression were assessed in aorta. Aortic lysates were resolved by SDS-PAGE. (**A**) Representative cropped western blot of total VASP and pVASP protein levels in aortas of the different groups of arteries. (**B**) Average densitometric data for pVASP/total VASP ratio. Data are expressed as mean ± SE (n = 12). ***P < 0.001 *vs* Wistar group; ^ϕ^P < 0.05, ^ϕϕϕ^P < 0.001 *vs* GK group.

The phosphorylation of vasodilator-stimulated phosphoprotein (VASP) at Ser^239^ has been shown to be an indicator of bioactive NO and the activity of the NO-cGMP-protein kinase G signalling pathway^18^. To establish NO bioavailability, we measured phosphorylated vasodilator-stimulated phosphoprotein (pVASP) and total VASP in the aorta. The ratio of pVASP/tVASP in GK rats was significantly decreased compared to Wistar rats in aorta (Fig. 5A,B). Treatment with SFN, PM or both significantly increased pVASP/tVASP ratio in diabetic GK rats (GKS, GKP; GKSP; Fig. 5A,B). A similar profile was observed in mesenteric arteries (data not shown).

### Nrf2 levels in aorta and mesenteric arteries

**Figure 6:**
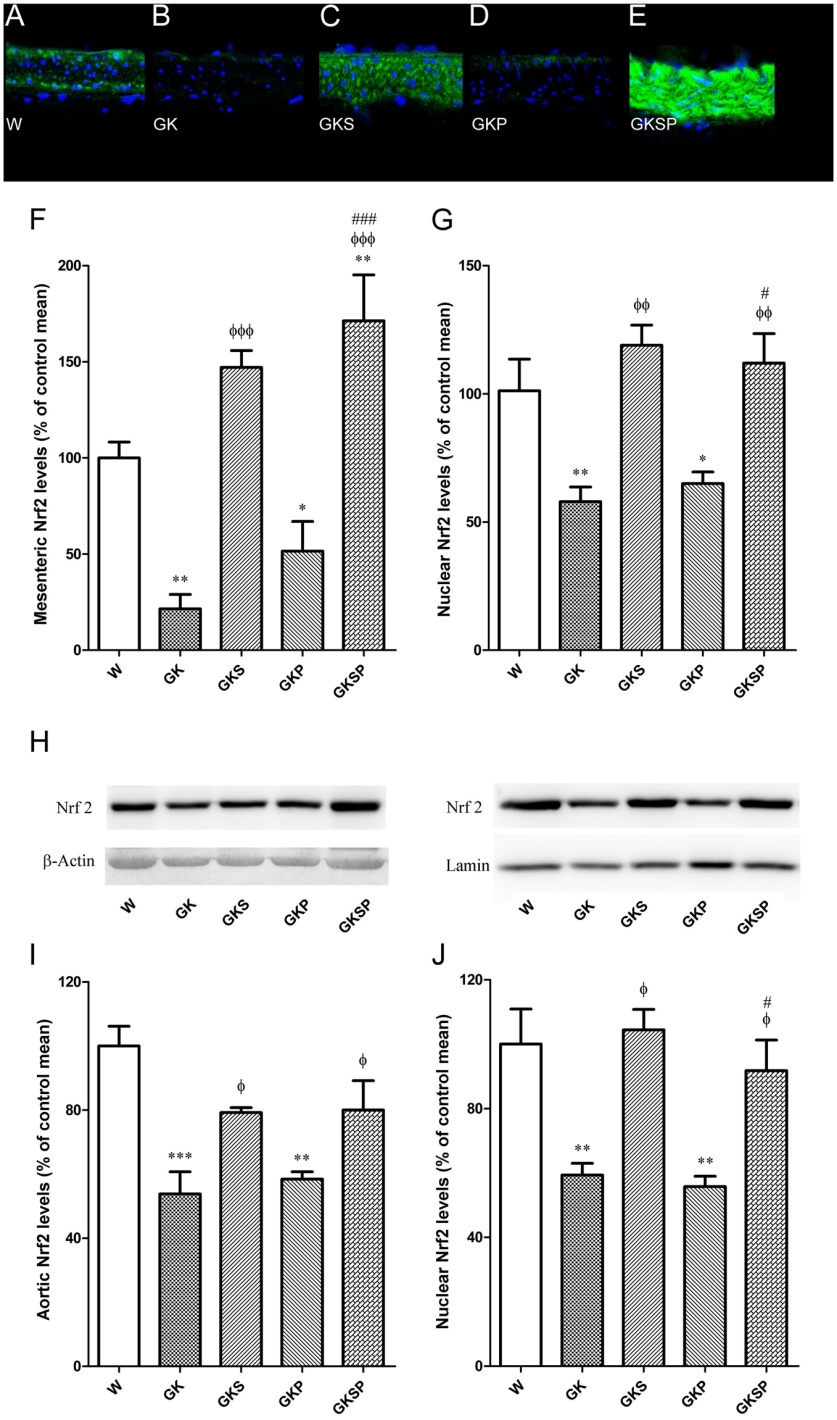
Effects of sulforaphane and pyridoxamine treatment on Nrf2 levels in aorta and mesenteric arteries of GK rats compared with nondiabetic Wistar rats. Representative mesenteric sections showing Nrf2 staining in nondiabetic Wistar (**A**), diabetic GK (**B**), and diabetic GK rats treated with SFN (GKS, **C**), PM (GKP, **D**) and SFN + PM (GKSP, **E**). Panel (F) contains quantification of the green fluorescence in the different groups of arteries. (**G**) The fluorescence intensity of Nrf2 label (green) localized to nuclear regions, as defined by DAPI staining (blue), was quantified and expressed as % of control mean of Wistar rats pooled from multiple mesenteric artery sections. (**H**) Cropped western blot of total Nrf2 (left panel) and nuclear Nrf2 protein levels in aortas of the different groups of arteries. Average densitometric data for total Nrf2 levels (**I**) and nuclear Nrf2 levels (**J**) in aortas normalized to the respective loading control. Data are mean ± SE. *P < 0.05, **P < 0.01, ***P < 0.001 vs Wistar group; ^ϕ^P < 0.05, ^ϕϕ^P < 0.01, ^ϕϕϕ^P < 0.001 *vs* GK group; ^#^P < 0.05, ^###^P < 0.001 *vs* GKP group.

We next quantified Nrf2 levels in the arteries of the different groups of rats. Nrf2 levels were assessed by western blot in aorta and by immunofluorescence with confocal microscopy in mesenteric arteries. Nrf2 levels were significantly down-regulated in vessels from the diabetic rats compared with age-matched Wistar rats in both aorta and mesenteric arteries (Fig. 6I,F). Sulforaphane treated groups (GKS and GKSP) dramatically increased the nuclear Nrf2 levels in aorta and mesenteric arteries (Fig. 6J,G). The Nrf2-activator SFN increases nuclear localization of Nrf2 in diabetic GK rats which probably leads to an increment in antioxidant defense promoting a decrement in vascular oxidative stress. SFN produced a significant increase in Nrf2 expression in both aorta and mesenteric arteries of diabetic GK rats (Fig. 6), suggesting that it promotes both increased expression and nuclear translocation, consistent with its documented effects in other tissues.

### AGE levels in aorta and mesenteric arteries

**Figure 7:**
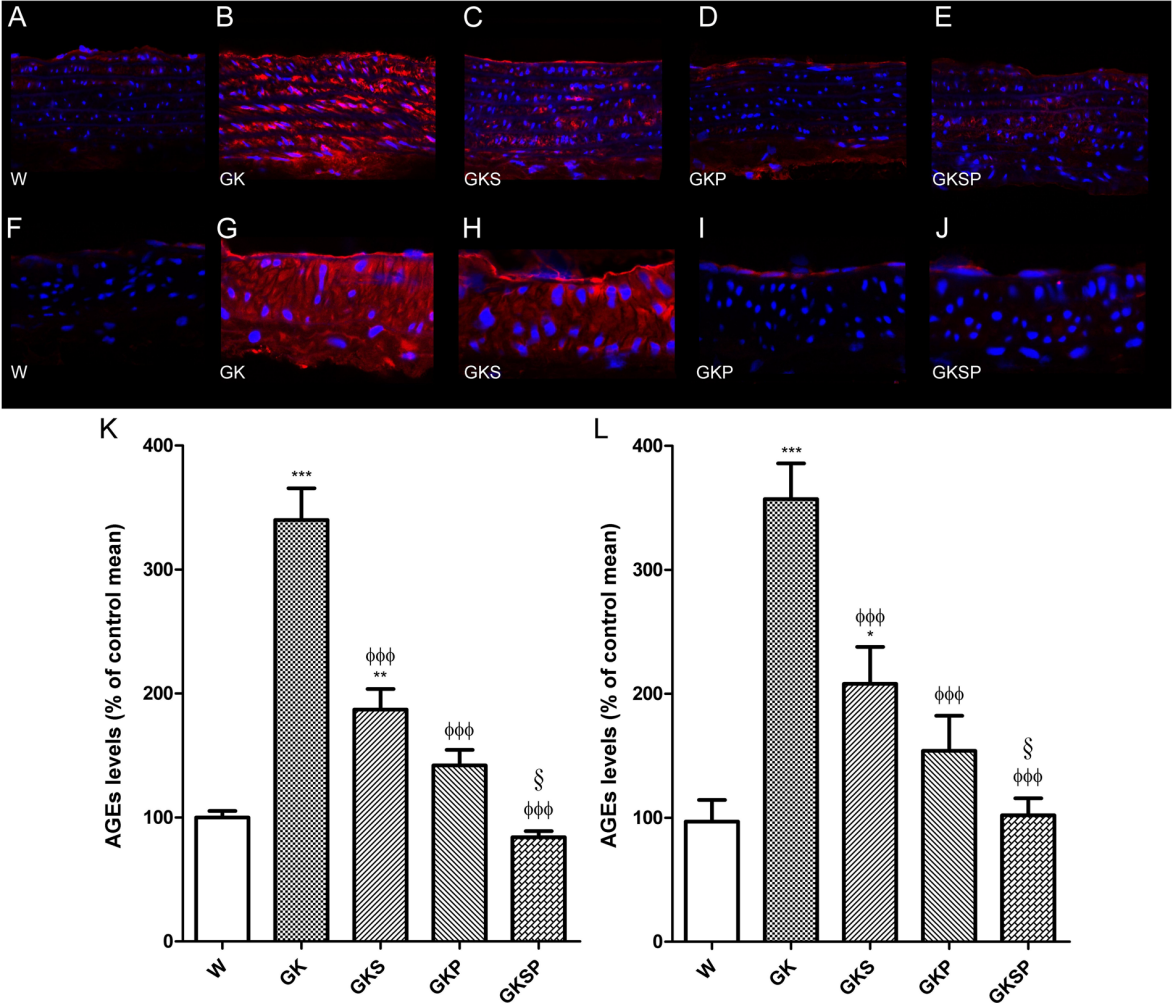
Effects of sulforaphane and pyridoxamine treatment on AGE levels in aorta and mesenteric arteries of GK rats compared with nondiabetic Wistar rats. Representative aortic sections showing AGE staining in nondiabetic Wistar (**A**), diabetic GK (**B**), and diabetic GK rats treated with SFN (GKS, **C**), PM (GKP, **D**) and SFN + PM (GKSP, **E**). Panel (K) contains quantification of the red fluorescence in the different groups of arteries. Representative mesenteric sections showing AGE staining in nondiabetic Wistar (**F**), diabetic GK (**G**), and diabetic GK rats treated with SFN (GKS, **H**), PM (GKP, **I**) and SFN + PM (GKSP, **J**). Panel (L) contains quantification of the red fluorescence in the different groups of arteries. Data are mean ± SE. *P < 0.05, **P < 0.01, ***P < 0.001 vs Wistar group; ^ϕ^P < 0.05, ^ϕϕ^P < 0.01, ^ϕϕϕ^P < 0.001 *vs* GK group; ^§^P < 0.05 *vs* GKS group.

We next quantified AGE levels in the arteries of the different groups of rats. AGE levels were assessed by immunofluorescence with confocal microscopy in aorta and mesenteric arteries. AGE levels were significantly increased in vessels from the diabetic rats compared with age-matched Wistar rats in both aorta and mesenteric arteries (Fig. 7B,K,G,L, respectively). GK treated groups (GKS, GKP and GKSP) significantly decreased the vascular AGE levels in aorta and mesenteric arteries (Fig. 7K,L). Noteworthy the groups treated with PM (GKP, GKSP) had normalized AGE levels in the vascular wall.

## Discussion

In the current study, the therapeutic potential of sulforaphane, pyridoxamine and its association is clearly demonstrated in diabetic endothelial dysfunction of GK rats. Treatment of GK rats with SFN, PM or both in association significantly reduced endothelial dysfunction in aorta and mesenteric arteries. In these vessels, SFN, PM or SFN + PM treatment retarded pathological characteristics of diabetic endothelial dysfunction including oxidative damage. Additionally, both therapies in association were able to reduce FFA levels and also increased NO bioavailability which can partially explain the normalization of endothelial function achieved in this long-term model of type 2 diabetes.

GK rats are an animal model of non-obese type 2 diabetes with mild hyperglycaemia, hyperinsulinaemia and insulin resistance^5,6,19^. Long-term diabetes is frequently associated with loss of pancreatic function in these animals, characteristics that also resemble features associated with human disease progression. This model shares many cardiovascular phenotypes with human type 2 diabetes including abnormal vascular reactivity^5,6^ justifying its use as a model to study diabetic complications.

Herein, we show that 8 months old GK rats are hyperglycaemic, with significantly increased total cholesterol and circulating levels of FFA. Endothelial dysfunction is evident in both mesenteric and aorta arteries accompanied by increased oxidative stress, glycation, decreased NO bioavailability and depletion of Nrf2 levels.

Nrf2 antioxidant functions may be important in vascular disease^20–22^. SFN, an activator of Nrf2 found in broccoli, increases the expression of protective enzymes under antioxidant response element-linked transcriptional control and prevents metabolic dysfunction in endothelial cells induced by hyperglycaemia^11^. The concentration of SFN used was determined based on other studies^23,24^ and on the bioavailability of this compound in broccoli sprouts. We estimate that SFN could have potential therapeutically benefits for the treatment of endothelial dysfunction at physiological doses feasible to be obtained in human diet^25^. On the other hand, we have recently examined the AGE levels in type 2 diabetes animal models and found that AGE levels are increased in plasma as well as in the vascular wall^26^. These high levels of AGE can be reduced with compounds such as PM and this approach has potential beneficial effects in endothelial function^17^. The therapeutic effect of both therapies at the macrovascular disease has not been previously studied. Herein, we study the effect of SFN with or without the concomitant inhibition of AGE formation with PM at the vascular level.

Treatment with SFN or PM alone for 8 weeks did not significantly improve the triglyceride, cholesterol and FFA levels. PM has been shown to correct hyperlipidaemia and nephropathy in both diabetic and obese rats, probably by interfering with various reactive carbonyl intermediates of AGE/advanced lipoxidation end products (ALE) formation from lipid oxidation^27,28^. This lipid-lowering effect was described for higher concentrations of PM (400 mg/Kg/day) in different animal models. Additionally, the Nrf2 pathway was identified as having regulatory functions in liver lipid metabolism. Nrf2 negatively regulates many genes encoding enzymes involved in lipid biosynthesis, fatty acid desaturation, and fatty acid transport^29^. In contrast, 0.5 mg/Kg of SFN impaired hepatic function and aggravated cholesterol levels in streptozotocin-induced diabetic rats^24^. Moreover in our experimental conditions SFN treatment alone did not change the lipid profile.

SFN + PM treatment had no effects on total cholesterol and triglycerides levels but it did significantly lower FFA and HbA1c levels. Both therapies in association were able to improve glucose intolerance, significantly reducing the glucose area under the curve. Targeting glycation and antioxidant defense systems with PM and SFN is highly effective in reducing FFA and HbA1c levels. Importantly, SFN and PM treatment had no effects on insulin levels, explaining the hyperglycaemia observed in the treated diabetic rats. Indeed, in all the GK treated groups, the plasma glucose levels were significantly higher than those of Wistar rats. However, the vasculoprotective actions of SFN + PM were evident under these conditions suggesting that the complete restoration of normoglycaemia is not a prerequisite for vasculoprotection.

Others have previously reported that activation of Nrf2 lowers plasma glucose levels and reduces diabetes-related nephropathy in wild type but not Nrf2 deficient mice^12^. In this study, SFN treated group (GKS) did not promote a decrement in blood glucose levels. Different animal models and experimental conditions may explain these differences.

The role of oxidative stress in the pathogenesis of endothelial dysfunction is well established. Diabetes increases ROS in the vasculature, compromises the antioxidant defense enzymes, and attenuates the levels of intracellular antioxidants^30^, creating an environment with increased oxidative stress^2,31,32^. In vascular models, activators of the Nrf2 pathway have been shown to restore redox homeostasis by increasing antioxidant/electrophilic response element-mediated expression of phase II and antioxidant enzymes, including NAD(P)H:quinone oxidoreductase-1, heme oxygenase-1 and γ-glutamate cysteine ligase catalytic subunit^33,34^. In this study, we present evidence that treatment with SFN and PM exerts anti-oxidative effects *in vivo*: the amounts of urinary 8-OHdG and tissue O_2_^• −^ anion and nitrotyrosine accumulation were diminished by SFN, PM or SFN + PM in diabetic GK rats. This anti-oxidative effect is an important factor behind the normalization mechanism of endothelial dysfunction observed. Accordingly, other studies have reported the inhibitory effect of SFN on oxidative stress under various conditions^10,35–37^. Targeting the Nrf2 pathway with SFN effectively restores normal redox balance and myogenic responsiveness to resistance arterioles in the diabetic db/db microvasculature^38^. In addition, SFN downregulates the expression of vascular cell adhesion molecule-1 in aortas of mice fed with a high fat diet mediated by an upregulation of Nrf2 activity^39^. Our data support a model in which oxidative stress is increased in aorta and mesenteric arteries by a mechanism involving an Nrf2-dependent reduction in the antioxidant capacity. We demonstrate, *in vivo*, that targeting the Nrf2 pathway with SFN effectively restores normal redox balance and ameliorates endothelial responsiveness to acetylcholine in aorta and mesenteric arteries in the diabetic vasculature. These beneficial effects are potentiated with PM, an inhibitor of AGE/ALE and cross-linking formation.

In diabetic animals, oxidative stress is elevated in proportion to the accumulation of AGE^40,41^ leading to the progression of vascular complications^42^. PM has been shown to inhibit AGE formation and the formation of lipid derived Maillard products; ALE^43^. It does not directly interact with Amadori products, but interferes with the post-Amadori oxidative reactions. Furthermore, PM traps reactive carbonyl compounds, inhibiting AGE and ALE adducts. PM inhibits lipaemia and the development of renal and vascular complications in obese rats^28^. The reduction of oxidative stress by PM may partly be due to inhibition of glycation - a process that directly causes free-radical production.

Moreover, treatment with SFN, PM or both significantly decreased vascular oxidative stress, and concomitantly increased NO bioavailability which can partially explain the beneficial effects on vascular function. AGE directly block NO activity and produce ROS in vascular endothelium^44^. The diabetic milieu is characterized by low levels of NO at the vascular level due to oxidative stress and increased AGE levels^2^. SFN and PM are highly effective in decrementing oxidative stress normalizing NO bioavailability. Probably only when AGE and ALE levels and cross-linking formation are lowered by PM together with the increment in antioxidant defense enzymes (SFN action) it is possible to promote higher levels of NO in the vasculature.

The effects of SFN on Nrf2-dependent gene expression are well documented^45^. Importantly, GK rats show a depletion of Nrf2 at the vascular level. In accordance, reduced levels of Nrf2 have been observed in mice diabetic arteries and human cardiac tissues^38,46–48^. Additionally, previous studies have suggested that aging is associated with Nrf2 dysfunction in the vasculature, which likely exacerbates age-related cellular oxidative stress and increases sensitivity of aged vessels to oxidative stress-induced cellular damage^49^. In agreement with these findings, we also found that Nrf2 expression in the aorta and mesenteric arteries were significantly down-regulated in the diabetic GK rats with 8 months old along with a significant nuclear decrement. The mechanism of Nrf2 reduction is not known and its elucidation is beyond the scope of the current study; nevertheless, our data demonstrate that disruption of Nrf2 signalling occurs in the diabetic vasculature and it probably leads to GSH depletion and decrement in other antioxidant defense mechanisms. In addition, previous studies have shown that Nrf2 induces the expression of glyoxalase 1 which protects against AGE induced protein and DNA damage and preserves cell function^50^.

Exploiting Nrf2 activators in association with inhibition of AGE/ALE levels and cross-linking formation and characterizing their beneficial effects may provide a novel therapeutic strategy for restoring cellular homeostasis in diabetes and its vascular complications. Presumably these findings lead to the discovery and evaluation of new antioxidant molecules, such as Nrf2 activators and AGE inhibitors, which may, at an early stage, hopefully inhibit the mechanism leading to diabetic complications.

## Conclusions

Collectively, our findings indicate that the therapeutic benefit of SFN + PM in endothelial dysfunction is multifactorial. In addition to its antioxidant function reducing oxidative stress, Nrf2 positively regulates NO bioavailability. These results provide convincing experimental evidence that SFN can be used with or without association with PM therapeutically to improve endothelial dysfunction and relieve vascular damage induced by diabetes. Decreasing glycation concomitantly with the activation of Nrf2 normalized endothelial function in an animal model with long-term diabetes highlighting the importance to target different mechanisms to achieve major beneficial outcomes.

Putting this together, one could envision a future strategy consisting not only in an early aggressive treatment of hyperglycaemia, but with the simultaneous use of compounds active on AGE formation, together with compounds capable of specifically targeting reactive species or capable of enhancing our antioxidant defense systems. Potential therapeutic effects of the simultaneous activation of Nrf2 and inhibition of AGE/ALE levels and cross-linking formation could be a potential strategy.

## Methods

### Drugs

PM, phenylephrine, acetylcholine, N-nitro-L-arginine- methyl ester (L-NAME), and polyethylene glycol-superoxide dismutase were obtained from SIGMA (St. Louis, MO, USA). Vasodilator-stimulated phosphoprotein (VASP), pVASP, β-actin, Nrf2, AGE (clone No. 6D12) and nitrotyrosine were obtained from Cell Signaling Technology (Danvers, MA, USA), Abcam plc (Cambridge, UK), Santa Cruz Biotechnology (Santa Cruz, CA, USA), Upstate Biotechnology (Lake placid, NY, USA) and Transgenic Inc (Kobe, Japan), respectively. Sulforaphane (SFN, L-isomer) was obtained from LKT laboratories (St. Paul, MN) whereas DHE was obtained from Invitrogen (Barcelona, Spain). All other chemicals and reagents used in the study were of high grade.

### Experimental animals

Wistar and GK rats were obtained from our local breeding colony (Faculty of Medicine, University of Coimbra, Portugal). Wistar and GK diabetic rats were divided into four experimental groups each. (1) Groups (W; GK) maintained with vehicle (corn oil) for 8 weeks; (2) Groups treated with sulforaphane (1 mg/Kg/day, intraperitoneal) for 8 weeks (WS; GKS); (3) Groups treated with pyridoxamine (100 mg/Kg/day, in the drinking water as previous^51^) for 8 weeks (WP; GKP); (4) Groups treated with sulforaphane and pyridoxamine for 8 weeks (WSP; GKSP). The animals were maintained with a standard commercial pellet chow and used with 8 months old. A pilot study (data not shown) with 3 different concentrations of SFN (0.5, 1 and 2 mg/Kg) has shown that 1 mg/Kg has a better outcome in terms of metabolic and endothelial function and it approaches the physiologic concentrations achieved by broccoli sprout extract ingestion in human and mice^25,52^.

All animals received care in accordance with the Portuguese Law on Experimentation with Laboratory Animals (last amendment, 2004), which is based on the principles of laboratory animal care as adopted by the *EU Directive 2010/63/EU for* animal experiments. The experimental protocols were approved by ORBEA – IBILI, Faculty of Medicine University of Coimbra.

### Determination of metabolic and oxidative stress parameters

After a 15 h fast, animals were anesthetized with ketamine/chlorpromazine [Ketamine chloride (75 mg/kg, im, Parke-Davis, Ann Arbor, MI, USA) and chlorpromazine chloride (2.65 mg/kg, im, Lab. Vitória, Portugal)] and killed by decapitation. Blood was taken by heart puncture for determination of lipids. Fasting serum lipids (total and HDL cholesterol and triglycerides) were quantified using commercially available kits Olympus-Diagnóstica Portugal, Produtos de Diagnóstico SA, Portugal). Plasma free fatty acids (FFA) levels were evaluated using enzymatic assay kits (Roche Applied Science, Portugal). Rats were placed in metabolic cages for 24 h and urine collected. Urinary 8-hydroxy-2-deoxyguanosine was measured using EIA kits (Cayman Chemical, Europe).

For glucose tolerance tests, rats were previously fasted overnight and were given an intraperitoneal injection of glucose (1.75 g kg^−1^ body weight). Blood glucose was determined by sampling from the tail vein at 0, 30, 60 and 120 min after glucose injection by a glucose-oxidase method using a glucometer (Glucometer-Elite-Bayer, Portugal S.A.) and compatible reactive test strips. The AUC of IPGTT curves was calculated with standard computer programs (GraphPad Prism PC Software version 3.0). Glycated haemoglobin (HbA1c) was determined using the A1C NOW + system (Bayer, Portugal S.A.).

### Isometric tension studies

Aorta were rapidly excised and freed of connective tissue. The aorta was divided into two segments (4-mm width). Ring segments were mounted between stainless steel triangles into individual organ chambers filled with oxygenated (95% O_2_, 5% CO_2_) modified Krebs-Henseleit buffer (37 °C, pH 7.4) (composition in mM: NaCl 119; KCl 4.7; CaCl_2_ 1.6; MgSO_4_ 1.2; NaHCO_3_ 25; KH_2_PO_4_ 1.2; Glucose 11.0). Indomethacin in a concentration of 10 µM was present in the experiments to inhibit prostaglandin synthesis. Aortic rings were subject to a resting tension of 9.8 mN. After equilibration for 60 min all vessels were preconstricted with phenylephrine. Ligand stimulated receptor-mediated NO bioavailability was assessed by a concentration-dependent relaxation to acetylcholine (ACh, 10^−9^ to 10^−3^ M), whereas sodium nitroprusside (SNP, 10^−9^ to 10^−4^ M) was used as an endothelium-independent agonist. Relaxation responses to ACh and SNP were expressed as percentage of relaxation from a submaximal phenylephrine-induced constriction and dose-response curves were obtained as previously described^5,6^.

Segments of second order superior mesenteric artery (approx. 200 μm external diameter) were trimmed free of fat and adhering connective tissue and mounted in a myograph according to the technique of Mulvany and Halpern^53^. Isometric tension was recorded and collected by a PowerLab data acquisition system (ADInstruments, UK) and recorded on a computer using the LabChart 7 data acquisition and analysis software (ADInstruments, UK). Segments were allowed to equilibrate for 30 min in modified Krebs-Henseleit solution, maintained at 37 °C and gassed with 5% CO_2_. Each vessel was placed under a stretch equivalent to the *in vivo* arterial blood pressure in each individual rat according to the technique of Mulvany and Halpern^53^. The tissue contractility was assessed by exposure to KCl (125 mM); subsequently cumulative concentration-response curves were obtained to phenylephrine (0.1–30 μM). Tissues were allowed to re-equilibrate for 30 min. The mesenteric rings were then used to study the response to one of the following agents: relaxation was assessed in response to either acetylcholine (ACh, 10^−9^ to 10^−6^ M) [both in the presence and absence of indomethacin (10 μM)] or sodium nitroprusside (SNP, 10^−9^ to 10^−3^ M); relaxation responses were obtained following preconstruction with phenylephrine to approximately 50% of the maximum as determined from the phenylephrine response curve as previous^54^. In a second series of experiments using mesenteric artery segments the acute effects of nitric oxide synthase inhibition were assessed using L-NAME. Responses to acetylcholine were obtained as above and subsequently repeated in the presence of L-NAME (300 μM). Relaxation responses to ACh and SNP were expressed as percentage of relaxation from a submaximal phenylephrine-induced constriction and concentration–response curves were obtained as previously described^54^.

### Detection of Superoxide

Unfixed frozen, 30-µm-thick sections of proximal aorta were incubated with DHE (2 × 10^−6^ M) in PBS for 30 minutes at 37 °C in a humidified chamber protected from light. DHE is oxidized on reaction with O_2_·^–^ to ethidium bromide, which binds to DNA in the nucleus and fluoresces red^6^. For ethidium bromide detection, images were obtained with a fluorescence microscope (Leica DMIRE200, Wetzlar, Germany). Fluorescence was detected with a 568-nm filter. Normal and diabetic tissues were processed and imaged in parallel with identical settings. Microscope and camera settings were kept constant for all preparations. Fluorescence was quantified using ImageJ (1.40 g, NIH).

### Assessment of mesenteric immunofluorescence

Sections (6 μm) of mesenteric arteries were washed with PBS and fixed in ice cold acetone, for 10 min. Sections were then permeabilized for 10 min in 1% Triton X-100 in PBS, pH 7.4, and blocked with 10% goat serum, for 30 min. Primary antibodies were diluted in PBS containing 0.02% BSA (PBS/BSA). The primary antibodies were added and the sections were incubated overnight at 4 °C. After incubation, the sections were extensively washed with PBS/BSA solution. After, sections were incubated with the secondary antibodies, diluted in PBS/BSA for 1 h. The coverslips were washed before mounting with Glycergel Dako mounting medium (Dako, Carpinteria, CA, USA). Immunostained mesenteric sections were visualized with a Leica DMIRE200 fluorescence microscope. Immunostained mesenteric sections were counterstained with DAPI and examined, photographed and quantified as described above for DHE fluorescence^54^.

### Western blot analysis

Segments of endothelium-intact vessels were washed with cold PBS and chilled in buffer containing in mM: Tris–HCl 50, NaCl 150, ethylenediamine tetraacetic acid (EDTA) 1, ethylene glycol tetraacetic acid (EGTA) 0.1, as well as NP-40, 0.1%, SDS 0.1% and deoxycholate 0.5%. Phenylmethylsulfonyl fluoride (1 mM), aprotonin (10 μg ml^−1^), leupeptin (10 μg ml^−1^), and pepstatin (10 μg ml^−1^) all from Sigma Chemicals (St. Louis, MO, USA) were added as the protease inhibitors. Tissues were homogenized in a standard fashion, followed by centrifugation at 14,000 × g for 20 min at 4 °C. The supernatants were collected and total protein concentration was determined. Samples containing 40 μg of protein were loaded on to a 12% sodium dodecyl sulfate-polyacrylamide gel electrophoresis (SDS-PAGE) gel, run and electroblotted onto polyvinylidene difluoride membrane. Prestained molecular weight marker proteins were used as standards for the SDS-PAGE. A ponceau staining was performed to verify the quality of the transfer and to ensure equal protein loading. Blots were blocked in 5% skimmed nonfat milk in PBS for 1 h, treated overnight with primary antibody against VASP, pVASP, β-actin or Nrf2 and then incubated with alkaline phosphatase secondary antibodies for 1 h. Anti-VASP phosphoserine 239 antibody was used for the analysis of the phosphorylation state of VASP at Ser239 (pVASP), which is a reliable biochemical marker of vascular cGMP-dependent protein kinase-1 activity. Activation of VASP was indicated by the intensity ratio pVASP/tVASP. Immunoblots were developed with an ECF Western blotting detection system (Amersham Biosciences).

Protein content was determined using a Bio-Rad protein assay kit.

### Statistical analysis

All data were analysed by standard computer programs (GraphPad Prism PC Software version 3.0, ANOVA) and are expressed as mean ± SEM (n = 12 individual animals per group). Significant differences were evaluated using one-way ANOVA followed by the Bonferroni post-hoc test for individual comparisons. P < 0.05 was considered significant. Dose response curves were fitted by nonlinear regression with simplex algorithm. Relaxation responses were given as the percentage of phenylephrine-preconstriction. Comparisons of dose–response curves were evaluated by 2-way ANOVA for repeated measures followed by the Bonferroni post-hoc test for individual comparisons.

## Electronic supplementary material

Supplement 1
