# Supplementary material for: The Sulforaphane and pyridoxamine supplementation normalize endothelial dysfunction associated with type 2 diabetes
Source: Sci Rep. 2017 Oct 30;7:14357. doi: 10.1038/s41598-017-14733-x (PMC5662716; doi:10.1038/s41598-017-14733-x)
Supplement: Supplementary file 1 — Supplement 1 [file 41598_2017_14733_MOESM1_ESM.pdf]

Sulforaphane and pyridoxamine supplementation normalize endothelial dysfunction associated with type 2 diabetes

Ana Pereira[1], Rosa Fernandes[2], Crisóstomo J[1], Raquel M. Seiça[1] and Cristina M. Sena[1*]

[1]Physiology, IBILI, [2]Ophthalmology, IBILI; Faculty of Medicine, University of Coimbra; Portugal

Address for reprint request and other correspondence:

[*]C. M Sena

Institute of Physiology, Sub-unidade 1, Pólo III

Faculty of Medicine, University of Coimbra

Azinhaga de Santa Comba, Celas

3000-504 Coimbra

Telephone: +351-239-480013

Fax: +351-239-480034

Email: csena @ ci.uc.pt

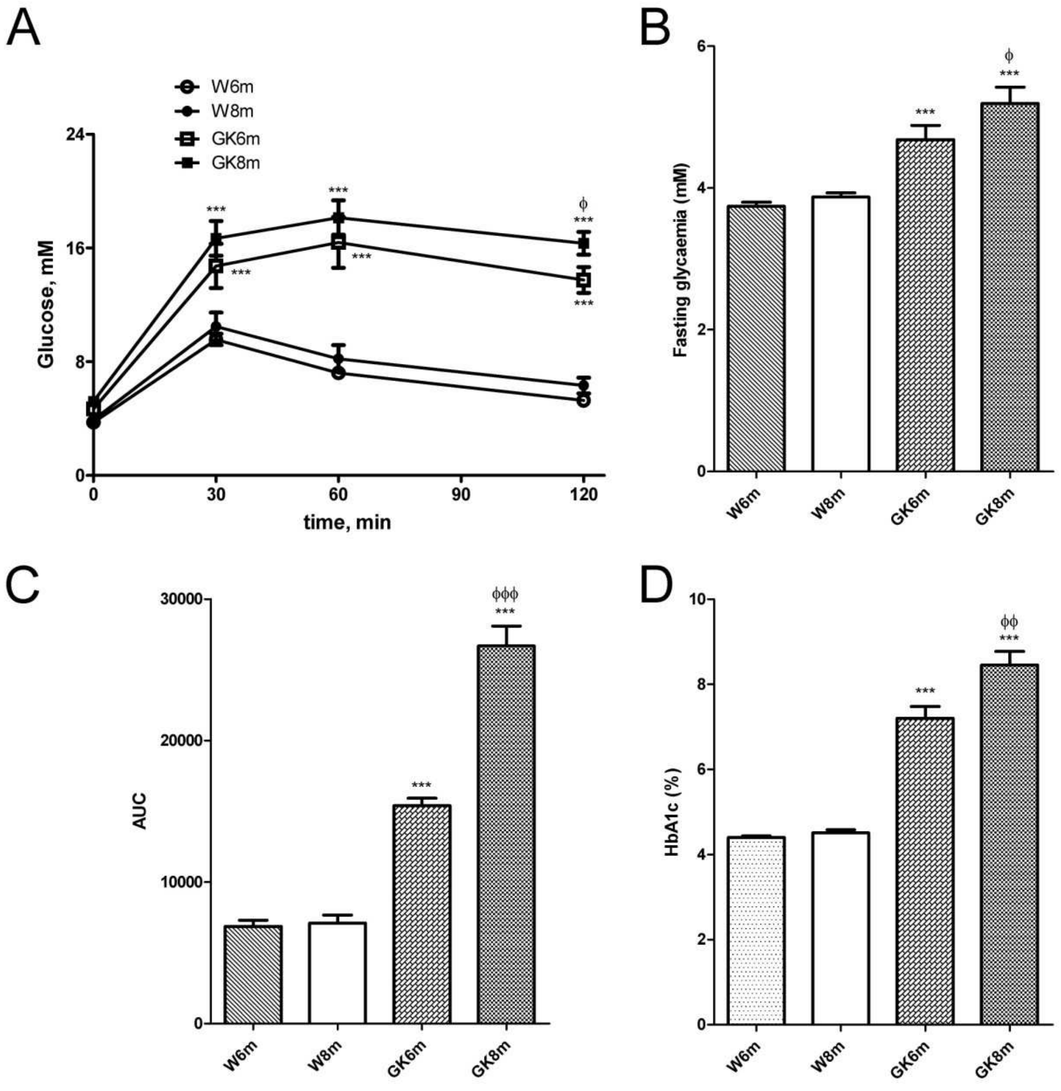

Figure 1 Intraperitoneal glucose tolerance test (IPGTT; A), fasting glycaemia (B), the glucose area under the curve (AUC; C) and HbA1c levels (D) in 8 months old diabetic Goto-Kakizaki (GK8m) rats compared with 6 months old GK rats (GK6m; before the treatments) and with age-matched Wistar (W8m and W6m) rats. C) The AUC of IPGTT curves was calculated to measure the degree of the glucose tolerance impairment. Data are expressed as mean±SE. *** P<0.001 *vs* age-matched Wistar group; ϕ P<0.05, ϕϕ P<0.01, ϕϕϕ P<0.001 *vs* GK6m group.

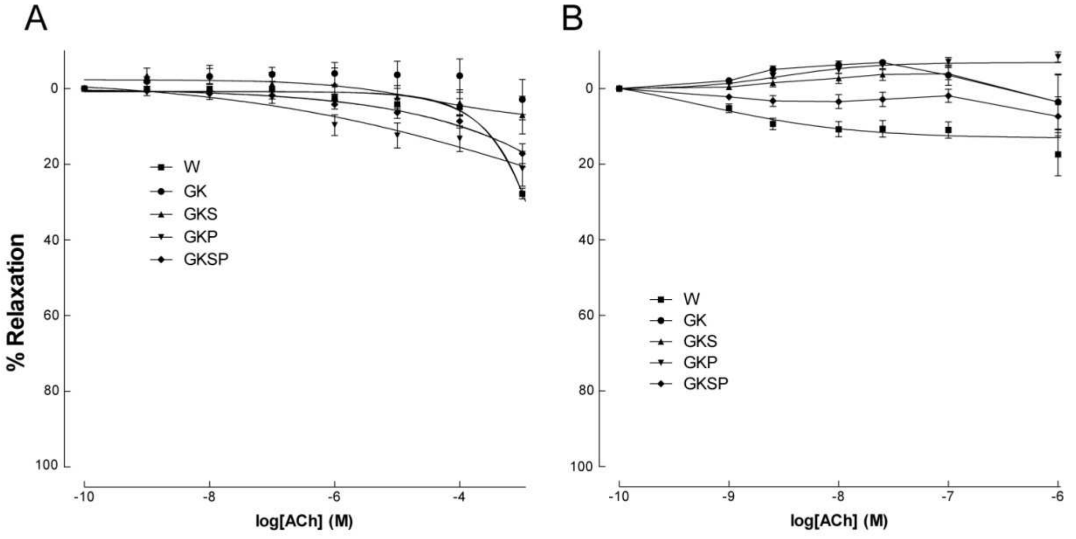

Figure 2 Effects of sulforaphane and pyridoxamine treatment on vasodilatory responses to acetylcholine in the presence of indomethacin (10μM) and N^{ω}-nitro-L-arginine methyl ester (L-NAME) 300 μM during 30 min after phenylephrine preconstriction of aortic segments in aorta (A) and mesenteric arteries (B) of GK rats compared with nondiabetic Wistar (W) rats. Indomethacin (10μM) was present throughout the experiments. Data are expressed as mean±SE (n=12).

Figure 6 Uncropped western-blots.

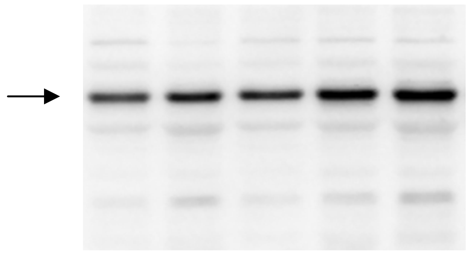

Total VASP

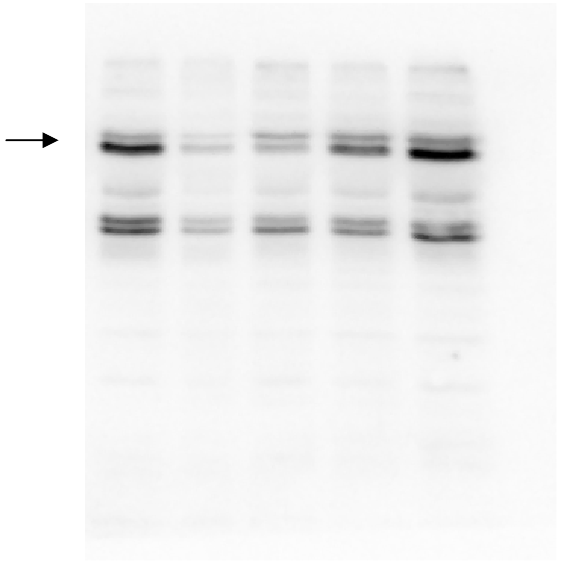

pVASP

Figure 7 Uncropped western-blots.

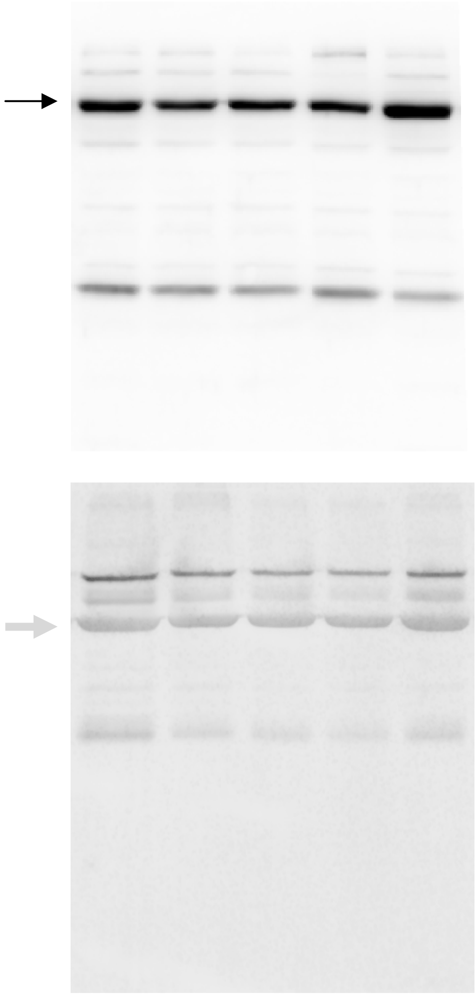

Nrf2 levels (black arrow); beta-actin (grey arrow).

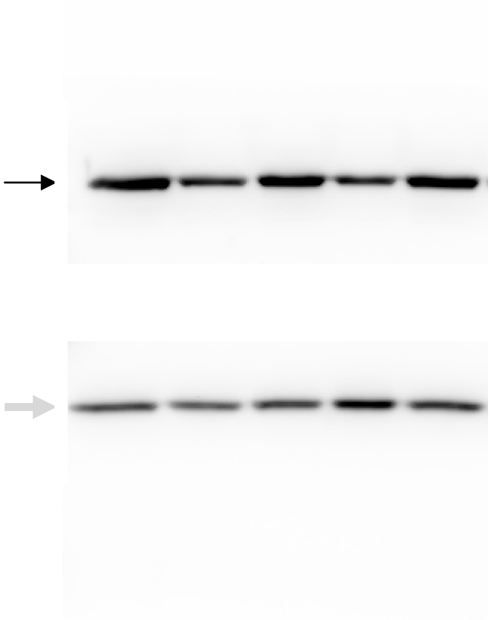

Nuclear Nrf2 levels (black arrow); lamin (grey arrow).
